# Experimental assessment of bioenergetic differences caused by the common European mitochondrial DNA haplogroups H and T

**DOI:** 10.1016/j.gene.2008.01.007

**Published:** 2008-03-31

**Authors:** Taku Amo, Nagendra Yadava, Richard Oh, David G. Nicholls, Martin D. Brand

**Affiliations:** a MRC Dunn Human Nutrition Unit, Hills Road, Cambridge CB2 0XY, UK; b Buck Institute for Age Research, Novato, California, USA

**Keywords:** *ρ*^0^, mtDNA-less, Δ*ψ*, mitochondrial membrane potential, TPMP, triphenylmethylphosphonium, FCCP, carbonyl cyanide *p*-trifluoromethoxyphenylhydrazone, Oxidative phosphorylation, Coupling efficiency, Sepsis, Sperm motility, Cybrid

## Abstract

Studies of both survival after sepsis and sperm motility in human populations have shown significant associations with common European mitochondrial DNA haplogroups, and have led to proposals that mitochondria bearing haplogroup H have different bioenergetic capacities than those bearing haplogroup T. However, the validity of such associations assumes that there are no non-random influences of nuclear genes or other factors. Here, we removed the effect of any differences in nuclear genes by constructing transmitochondrial cybrids harbouring mitochondria with either haplogroup H or haplogroup T in cultured A549 human lung carcinoma cells with identical nuclear backgrounds. We compared the bioenergetic capacities and coupling efficiencies of mitochondria isolated from these cells, and of mitochondria retained within the cells, as a critical experimental test of the hypothesis that these haplogroups affect mitochondrial bioenergetics. We found that there were no functionally-important bioenergetic differences between mitochondria bearing these haplogroups, using either isolated mitochondria or mitochondria within cells.

## 1 Introduction

Analysis of the variation in normal human mitochondrial DNA (mtDNA) has identified many haplogroups–specific patterns of polymorphisms that have arisen over the last 150,000–200,000 years–that have been used to study the origin, radiations and evolution of human populations (Cann et al., 1987; Wallace et al., 1999; Wallace, 2005). These mtDNA haplogroups have been less studied bioenergetically than mtDNA mutations that lead to disease (Dimauro and Davidzon, 2005; Taylor and Turnbull, 2005), but normal variation in mtDNA may affect disease susceptibility (Herrnstadt and Howell, 2004) and longevity (Santoro et al., 2006). These effects could be explained by differences in mitochondrial coupling efficiency (the percentage of oxygen consumption used for ATP synthesis rather than heat generation) or mitochondrial production of ROS (reactive oxygen species). It has been proposed that particular haplogroups cause inefficient oxidative phosphorylation and greater heat production and were therefore selected during the radiations of humans into Arctic environments (Mishmar et al., 2003; Ruiz-Pesini et al., 2004; Wallace, 2005; Montiel-Sosa et al., 2006), but this proposal is controversial (Elson et al., 2004; Kivisild et al., 2006; Ruiz-Pesini and Wallace, 2006) and is not supported by direct measurement of mitochondrial coupling efficiencies in mitochondria carrying representative “arctic” and “tropical” mtDNAs (Amo and Brand, 2007; Elson et al., 2007).

Two lines of evidence suggest that common European haplogroups may affect bioenergetic function: there are associations of both survival after sepsis and sperm motility with haplogroup H. In a prospective study of intensive care patients in Newcastle-upon-Tyne, U.K., individuals with haplogroup H (about 40% of the population studied) were more than twice as likely to survive 180 days after sepsis than those with non-H haplogroups (primarily the closely-related haplogroups J and T; about 30% of the population) (Baudouin et al., 2005). Mitochondrial dysfunction may be linked to sepsis-induced multiple organ failure (Protti and Singer, 2007), so altered mitochondrial bioenergetics might be the causal link between haplogroup and survival. Two possibilities have been proposed: haplogroup H might protect through greater heat generation (because of higher electron transport rates or looser coupling) (Baudouin et al., 2005), or through greater ROS production (because of tighter coupling and raised protonmotive force), which could reduce bacterial infection (Wallace, 2005). There have been no direct experimental tests of these possibilities.

The second line of evidence comes from studies of sperm motility, which is correlated with mitochondrial enzymatic activities and depends on the activity of the mitochondrial electron transport chain (Ruiz-Pesini et al., 1998; Ruiz-Pesini et al., 2000). Haplogroup T was over-represented in men with asthenozoospermia (reduced sperm motility), whereas haplogroup H was over-represented in men with other fertility problems. Sperm with haplogroup H performed better in a test of motility and had higher cytochrome oxidase activity than those with haplogroup T (Ruiz-Pesini et al., 2000), suggesting that mitochondria carrying haplogroup H make more ATP than those with haplogroup T. This conclusion has been extended to sublineages of haplogroup U (Montiel-Sosa et al., 2006), but associations between haplogroups and reduced male fertility and sperm motility have been disputed by others (Pereira et al., 2005; Pereira et al., 2007). Less well-coupled mitochondria make less ROS (Korshunov et al., 1997; Liu, 1997; Lambert and Brand, 2004), so people having such mitochondria might suffer less from neurodegenerative diseases caused by ROS (Wallace, 2005). Some epidemiological studies support this prediction; e.g. haplogroup T is under-represented in Alzheimer's disease patients (Chagnon et al., 1999). However, other studies have not replicated this finding (Elson et al., 2006), and correlations between mitochondrial haplogroups and neurodegenerative diseases are controversial (Raule et al., 2007). Again, there have been no direct experimental tests of possible differences in the bioenergetic properties of mitochondria that might underlie the reported associations of haplogroup and phenotype.

In the present study, we analyse bioenergetic capacity and coupling efficiency in mitochondria isolated from cytoplasmic hybrids (cybrids) carrying haplogroups H and T with identical nuclear DNA. Furthermore, to investigate the effects of haplogroups H and T on the bioenergetic status of mitochondria at the cellular level, we analyse the respiratory capacities and mitochondrial coupling efficiencies of intact cybrids.

## 2 Materials and methods

### 2.1 Subjects

Healthy volunteers were recruited by advertisement and written informed consent was obtained. Ethical approval was obtained from the Cambridge Research Ethics Committee. DNA was extracted from buccal swab samples using a QIAamp DNA Mini Kit (Qiagen, Hilden, Germany). Mitochondrial haplogroups were determined by PCR-RFLP analysis according to published criteria (Wallace et al., 1999). Entire mtDNAs were amplified in several overlapping fragments by PCR (Torroni et al., 1997). Each fragment was digested by restriction enzymes and resolved on agarose gels. 15 ml of blood were taken from each of three volunteers who had haplogroup H, and three who had haplogroup T, for platelet preparation and cybrid construction. Haplogroup H (Achilli et al., 2004) is subdivided into at least 15 sub-haplogroups (H1-H15). In the Newcastle sepsis study, there were no significant differences in survival of sub-haplogroups H1 (− 3008 TaqI), H2 (− 4769 AluI), H3 (+ 6773 NlaIII) and the remaining H sub-haplogroups combined (Baudouin et al., 2005). Our haplogroup H volunteers were H1, H3 and (H, not H1, H2 or H3). Haplogroup T (Macaulay et al., 1999) is subdivided into at least 5 sub-haplogroups (T1–T5) (Pike 2006). Our haplogroup T volunteers were one T (sub-haplogroup not checked), one T1 (− 12629 AvaII) and one (T, not T1). No major differences in cybrid phenotypes between sub-haplogroups were observed.

### 2.2 Generation of cybrid cell lines

Cybrid cell lines are constructed by repopulation of mtDNA-less (*ρ*^0^) cells with exogenous mitochondria (King and Attardi, 1989). A549.B2 *ρ*^0^ (mtDNA-less) cells derived from human lung carcinoma A549 (originally carrying mitochondrial DNA of haplogroup H) were cultivated in Dulbecco's modified Eagle's medium (DMEM) containing 4.5 g/l glucose, 110 µg/ml sodium pyruvate, 10% (v/v) fetal bovine serum (FBS) and 50 µg/ml uridine. Platelets (which have no nuclei) were isolated from the volunteers' blood samples and fused with A549.B2 *ρ*^0^ cells as described elsewhere (Chomyn, 1996). The resultant cybrids had mtDNA from the different donors, but their nuclear DNA was identical. After many generations of cybrid growth (diluting platelet-derived nuclear-encoded subunits), all nuclear-encoded mitochondrial protein subunits will be specified by the host cell, but all mitochondrial-encoded subunits will be specified by the donor mtDNA. The cybrid cell lines were constructed and routinely maintained in DMEM with 10% dialysed FBS. Stocks of cell lines were frozen and kept at − 80 °C until required. DNA was extracted from cultured cybrid cells as described previously (Laird et al., 1991) to confirm mitochondrial haplogroups by PCR-RFLP analysis.

### 2.3 Mitochondrial respiration and membrane potential

Human A549 cell mitochondria were prepared from cultured cybrids as previously described (Amo and Brand, 2007). Mitochondrial oxygen consumption was measured at 37 °C using a Clark electrode (Rank Brothers, Cambridge, UK) calibrated with air-saturated medium comprising 0.115 M KCl, 10 mM KH_2_PO_4_, 3 mM Hepes (pH 7.2), 2 mM MgCl_2_, 1 mM EGTA and 0.3% (w/v) defatted BSA, assumed to contain 406 nmol atomic oxygen·ml^− 1^ (Reynafarje et al., 1985). No correction was made for consumption of oxygen by the electrode, so oxygen consumption at low rates will be slightly overestimated. Mitochondrial membrane potential (Δ*ψ*) was measured simultaneously with respiratory activity using an electrode sensitive to the lipophilic cation TPMP^+^ (triphenylmethylphosphonium) (Brand, 1995). Mitochondria were incubated at 1.0 mg·ml^− 1^ (for succinate respiration) or 1.5 mg·ml^− 1^ (for 2-oxoglutarate + malate respiration) in the presence of 80 ng·ml^− 1^ nigericin (to collapse the pH gradient so that the protonmotive force was expressed exclusively as Δ*ψ*) and 4 µM rotenone (to inhibit complex I). The TPMP^+^-sensitive electrode was calibrated with sequential additions of TPMP^+^ up to 2 µM, then 4 mM succinate or 3.2 mM 2-oxoglutarate + 0.8 mM malate (with rotenone omitted) was added to initiate respiration. Experiments were terminated with 1.6 µM FCCP (carbonyl cyanide *p*-trifluoromethoxyphenylhydrazone), allowing correction for any small baseline drift. Δ*ψ* was calculated from the distribution of TPMP^+^ across the mitochondrial inner membrane using a binding correction factor of 0.35 mg protein·µl^− 1^. Respiratory rates and respiratory control ratios (the state 3 respiration rate with 0.8 mM ADP divided by the state 4 rate with oligomycin) with 2-oxoglutarate + malate as substrate were determined in the absence of nigericin.

### 2.4 Modular kinetics

To investigate differences in oxidative phosphorylation caused by mitochondrial DNA variants, we applied a systems approach: modular kinetic analysis (Amo and Brand, 2007). This analyses the kinetics of the whole of oxidative phosphorylation divided into three modules connected by their common substrate or product, Δ*ψ*. The modules are (i) the reactions that produce Δ*ψ*, consisting of the substrate translocases, dehydrogenases and other enzymes and the components of the respiratory chain, called ‘substrate oxidation’, (ii) the reactions that consume Δ*ψ* and synthesize, export and dephosphorylate ATP, consisting of the ATP synthase, the phosphate and adenine nucleotide translocases and any ATPases that may be present, called the ‘phosphorylating system’, and (iii) the reactions that consume Δ*ψ* without ATP synthesis, called the ‘proton leak’ (Brand, 1990). The analysis reports changes anywhere within oxidative phosphorylation that are functionally-important, but is unresponsive to changes that have no functional consequences. Comparison of the kinetic responses of each of the three modules to Δ*ψ* obtained using mitochondria isolated from different cybrids reveals any effects of mitochondrial haplogroup on the kinetics of oxidative phosphorylation. Oxygen consumption and Δ*ψ* were measured simultaneously using mitochondria incubated with 80 ng·ml^− 1^ nigericin and 4 µM rotenone. Respiration was initiated by 4 mM succinate or 3.2 mM 2-oxoglutarate + 0.8 mM malate (with rotenone omitted). The kinetic behaviour of a ‘Δ*ψ*-producer’ can be established by specific modulation of a Δ*ψ*-consumer and the kinetics of a consumer can be established by specific modulation of a Δ*ψ*-producer (Brand, 1998). To measure the kinetic response of proton leak to Δ*ψ*, the state 4 (non-phosphorylating) respiration of mitochondria in the presence of oligomycin (0.8 µg·ml^− 1^; to prevent any residual ATP synthesis), which was used solely to drive the proton leak, was titrated with malonate (up to 0.5 mM). In a similar way, state 4 respiration was titrated by FCCP (up to 0.8 µM) for measurement of the kinetic response of substrate oxidation to Δ*ψ*. State 3 (maximal rate of ATP synthesis) was obtained by addition of excess ADP (0.8 mM). Titration of state 3 respiration with malonate (up to 1.15 mM) allowed measurement of the kinetics of the Δ*ψ*-consumers (the sum of the phosphorylating system and proton leak). The coupling efficiencies of oxidative phosphorylation were calculated from the kinetic curves as the percentage of mitochondrial respiration rate at a given Δ*ψ* that was used for ATP synthesis and was therefore inhibited by oligomycin. Note that any slip reactions will appear as proton leak in this analysis (Brand et al., 1994).

### 2.5 Cell respiration

Cell respiration was measured at 37 °C using a Seahorse XF24 Extracellular Flux Analyzer (Seahorse Bioscience, Billerica, MA, USA), which is fully described elsewhere (Wu et al., 2007). Cybrids were thawed and subcultured in Petri dishes in DMEM to which had been added 4.5 g/l glucose, 110 µg/ml sodium pyruvate, 0.02 vol of penicillin/streptomycin (5000 units/5000 µg/ml), 0.02 vol of 200 mM l-glutamine and 0.1 vol Fetal Bovine Serum (heat inactivated). Cybrids were seeded at 20,000–50,000 cells per well in Seahorse XF24 24-well plates and grown for 3–5 d in a 37 °C incubator under 95% air/5% CO_2_, then washed and incubated for about 1 h in a 37 °C incubator under air in 700 µl of assay medium (comprising 120 mM NaCl, 3.5 mM KCl, 2 mM MgCl_2_, 1.3 mM CaCl_2_, 1.2 mM Na_2_SO_4_, 0.4 mM KH_2_PO_4_, 15 mM d-glucose and 0.4% (w/v) BSA, pH adjusted to 7.4 with NaOH). Experiments were carried out to a paired design: each plate was seeded half with one H-haplotype cybrid line and half with one T-haplotype cybrid line. Appropriate amounts of concentrated stocks of oligomycin, FCCP, rotenone and myxothiazol dissolved in 70% ethanol were diluted into 75 µl of assay medium and loaded into the appropriate addition ports in a measuring cartridge. The plate and measuring cartridge (containing waveguides and fluorescent oxygen sensors together with reagents for automated pneumatic additions of inhibitors and uncouplers) was loaded into the machine and after the calibration procedure (30 min calibration, 10 min rest), measurement cycles of 1 min sample mixing, 2 min waiting and 3 min measurement of oxygen consumption rate were initiated. All respiration rates were calculated as a percentage of the rate in the same well for the fourth measurement point — the basal rate just preceding addition of oligomycin (see Fig. 4). This corrected for differences in cell density between wells. In the range measured, absolute rates of oxygen consumption were linearly related to cell numbers seeded. Absolute basal oxygen consumption rates for the parental A549 cell line are reported to be about 1.7 nmol/min/10^6^ cells (Wu et al., 2007). Respiration rates at each time-point from three or four replicate wells were averaged.

### 2.6 Statistics

**Fig. 1:**
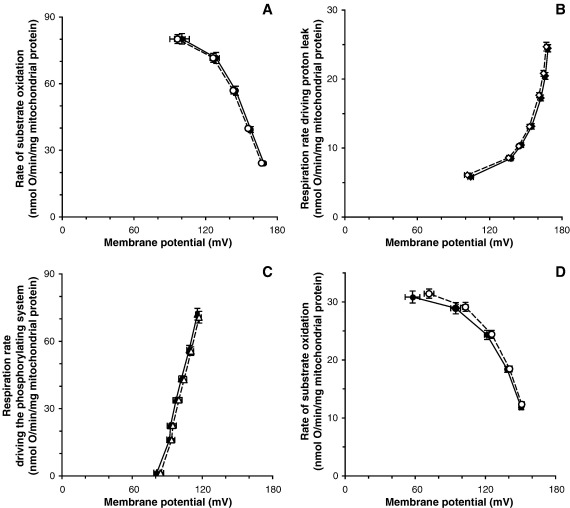
Modular kinetic analysis of oxidative phosphorylation in mitochondria isolated from cybrids. Modular kinetic analysis, using 4 mM succinate as substrate, of the kinetic responses to membrane potential, Δ*ψ*, of respiration driving (A) substrate oxidation (Δ*ψ* titrated with uncoupler, FCCP), (B) proton leak (Δ*ψ* titrated with malonate, starting in state 4) and (C) the phosphorylating system, calculated by subtracting respiration driving proton leak from respiration driving the Δ*ψ*-consumers (Δ*ψ* titrated with malonate starting in state 3; not shown) at each Δ*ψ*. (D) Kinetic response to Δ*ψ* of respiration driving substrate oxidation using 3.2 mM 2-oxoglutarate + 0.8 mM malate as substrate, with rotenone omitted. Closed symbols, haplogroup H; open symbols, haplogroup T. Results are means ± SEM (*n* = 15 cell clones — five different cybrid clones for each donor; three donors for each haplogroup). There were no significant differences between haplogroups H and T for any of the data in Fig. 1.

**Fig. 2:**
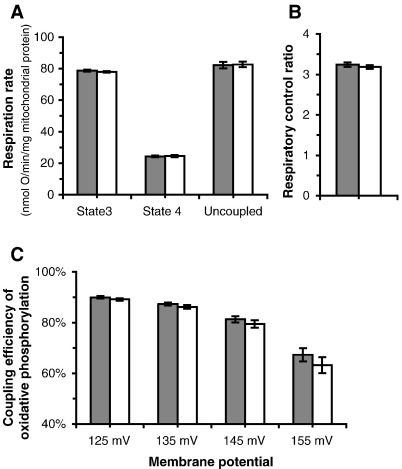
Respiration rates and coupling efficiency of oxidative phosphorylation using succinate as substrate in mitochondria isolated from cybrids. Closed bars, haplogroup H; open bars, haplogroup T. (A) State 3, state 4 and uncoupled respiration rates. (B) Respiratory control ratio (state 3 respiration rate/state 4 respiration rate). (C) Coupling efficiency at selected Δ*ψ* values between state 3 and state 4. Data are means ± SEM (*n* = 15 cell clones- five different cybrid clones for each donor; three donors for each haplogroup). Values were calculated from the results shown in Fig. 1. There were no significant differences between haplogroups H and T for any of the data in Fig. 2.

**Fig. 3:**
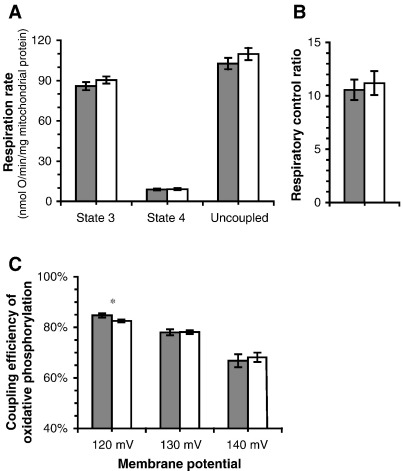
Respiration rates and coupling efficiency of oxidative phosphorylation using 2-oxoglutarate + malate as substrate in mitochondria isolated from cybrids. Closed bars, haplogroup H; open bars, haplogroup T. (A) State 3, state 4 and uncoupled respiration rates, all in the absence of nigericin. (B) Respiratory control ratio (state 3 respiration rate/state 4 respiration rate) in the absence of nigericin. (C) Coupling efficiency at selected Δ*ψ* values between state 3 and state 4 in the presence of nigericin. Data are means ± SEM (*n* = 15 cell clones — five different cybrid clones for each donor; three donors for each haplogroup). Values in (C) were calculated from the results shown in Fig. 1. H^+^/O ratios were taken to be 10 and 6 for respiration on 2-oxoglutarate + malate and succinate, respectively (Brand, 2005); the curves for proton leak with succinate as substrate (Fig. 1B) were scaled by 6/10 and used to calculate the coupling efficiencies with oxoglutarate + malate as substrate using the data in Fig. 1D. ^⁎^*P* < 0.05.

**Fig. 4:**
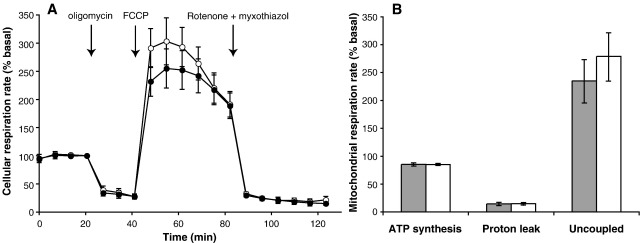
Cellular and mitochondrial respiration rates in intact cybrids. Closed symbols and bars, haplogroup H; open symbols and bars, haplogroup T. (A) Oxygen consumption rates of cells grown in 24-well plates measured using a Seahorse XF24 Extracellular Flux Analyzer; the time after the first measurement is indicated. Rates were expressed as a percentage of the basal rate in each well measured at about 20 min, just before oligomycin addition. At the times indicated, final concentrations of 1 µg/ml oligomycin, 3 µM FCCP and 1 µM rotenone plus 2 µM myxothiazol were injected automatically. (B) Mitochondrial respiration rates in intact cybrids calculated from (A). Non-mitochondrial oxygen consumption was calculated as the mean of the last three points in (A), and subtracted from the mean of the first four points in (A). The difference was scaled to give 100% of basal mitochondrial respiration. Respiration driving ATP synthesis was calculated as the mitochondrial rate sensitive to oligomycin, and respiration driving proton leak as the mitochondrial rate insensitive to oligomycin, both calculated from the mean of the three points after addition of oligomycin in (A). Uncoupled mitochondrial respiration was calculated arbitrarily as the mean of the first three points after addition of FCCP. Data are means ± SEM (*n* = 6 cell clones, two different cybrid clones for each donor and three donors for each haplogroup). There were no significant differences between haplogroups H and T for any of the data in Fig. 4.

Values are given as mean ± SEM for *n* cell clones (*n* = 15 cell clones for Figs. 1–3, five different clones for each donor and three donors for each haplogroup, and *n* = 6 cell clones for Fig. 4, two different clones for each donor and three donors for each haplogroup). To test for significant differences in modular kinetics between haplogroups, we interpolated the mean respiration rates of mitochondria from the individual clones at a range of different potentials between the highest and lowest common membrane potential. The significance of differences between means was assessed by unpaired Student's *t*-test using Microsoft Excel X; *P* values < 0.05 were taken to be significant.

## 3 Results

### 3.1 Modular kinetic analysis of oxidative phosphorylation

Haplogroup H is a subgroup of haplogroup HV, and haplogroup T is a subgroup of haplogroup JT; HV and JT diverged about 40,000 years ago (Kivisild et al., 2006). Haplogroup H is found in about 41% of Europeans and haplogroup T is found in about 15% (Wallace et al., 1999). Haplogroup H differs from haplogroup T by four non-synonymous mutations in proteins (one each in the complex I subunits, ND1 and ND2, and two in the complex III subunit, cytochrome b), two mutations in tRNAs (tRNA^arg^ and tRNA^thr^) and three mutations in rRNA (two in 16S rRNA and one in 12S rRNA) (Kivisild et al., 2006), so differences in oxidative phosphorylation between these haplogroups are plausible.

To compare mitochondrial functions between haplogroup H and T, we measured the kinetics and coupling efficiency of oxidative phosphorylation using mitochondria isolated from cybrid cells. Fig. 1A, B and C shows the kinetics of the three modules of oxidative phosphorylation using succinate as respiratory substrate. Fig. 1A shows the kinetic response of substrate oxidation to its product, Δ*ψ*; Fig. 1B shows the kinetic response of proton leak to its driving force, Δ*ψ*, and Fig. 1C shows the kinetic response of the ATP phosphorylating pathway to its driving force, Δ*ψ*. The three sets of kinetics curves using succinate were indistinguishable between mtDNA haplogroup H (closed symbols) and T (open symbols). We also measured the kinetics of substrate oxidation using 2-oxoglutarate + malate as substrate instead of succinate to check for any effects of differences in the mitochondrial complex I genes. Once again, the kinetic response of the substrate oxidation system to Δ*ψ* using 2-oxoglutarate + malate as substrate was not significantly different between haplogroups H and T (Fig. 1D).

From the kinetic curves shown in Fig. 1, we can analyse the coupling efficiency of oxidative phosphorylation. The classic measure is the respiratory control ratio: respiration at maximal rate of ATP synthesis (state 3) divided by respiration with no ATP synthesis (state 4) (Fig. 2A and B). A more precise measure of coupling efficiency is given by the percentage of respiration that is coupled to ATP synthesis. This is calculated by subtracting the rate of proton leak (Fig. 1B) from the total rate of respiration (Fig. 1A) at any chosen membrane potential, and expressing the difference as a percentage of the total rate. If this percentage is lower, then a greater proportion of the redox energy is diverted to heat production. Mitochondria in cells normally operate at less than maximal rates and values of Δ*ψ*. Fig. 2C reports the coupling efficiency across the whole range of rates of ATP synthesis and values of Δ*ψ* from state 3 (maximal rate of ATP synthesis) to close to state 4 (where ATP synthesis is minimal). As expected (Brand et al., 1993), coupling efficiency decreased as mitochondria moved from state 3 towards state 4. There were no significance differences in rates, respiratory control ratio or coupling efficiencies between haplogroups H and T.

We also independently measured the respiratory control ratio using 2-oxoglutarate + malate as substrate and calculated coupling efficiency from the data in Fig. 1 (Fig. 3). Once again, there was no clear difference between haplogroup H and T, either in respiratory rates (Fig. 3A), respiratory control ratio (Fig. 3B) or coupling efficiencies across a range of rates and potentials (Fig. 3C).

In summary, at the mitochondrial level we observed no significant functional differences between mitochondria isolated from cybrids of haplogroup H and haplogroup T.

### 3.2 Coupling efficiency of respiration in intact cybrid cells

To check whether differences in mitochondrial function between haplogroups were lost when mitochondria were isolated from the cells and measured at a standardised protein concentration in an artificial incubation medium, we measured the respiration rates and coupling efficiencies of mitochondria in intact cybrids. Cybrids were grown on 24-well plates and oxygen consumption was measured using a Seahorse XF24 Extracellular Flux Analyzer. Fig. 4A shows that oxygen consumption was inhibited when ATP synthesis was prevented by addition of oligomycin, stimulated when oxidation was uncoupled from phosphorylation by addition of the uncoupler FCCP, and inhibited again when mitochondrial electron transport was abolished by addition of rotenone (to inhibit complex I of the electron transport chain) and myxothiazol (to inhibit complex III).

The residual rate in the presence of rotenone and myxothiazol represents non-mitochondrial oxygen consumption. This comprised 16.7 + 2% of basal respiration rate in H-haplotype cybrids and 18.2 + 2.7% in T-haplotype cybrids. These values were not significantly different. Subtraction of this non-mitochondrial respiration allowed assessment of the rates and coupling efficiency of the remaining mitochondrial respiration.

Oligomycin inhibits mitochondrial ATP synthesis, so mitochondrial respiration that is insensitive to oligomycin is due to uncoupled respiration, i.e. to proton leak pathways through the mitochondrial inner membrane, and respiration that is sensitive to oligomycin is due to ATP synthesis occurring in the cells. Because oligomycin prevents the energy-utilizing pathway of ATP synthesis, the mitochondrial proton motive force will rise after oligomycin addition, and the proton leak rate will increase, leading to an overestimate of the proton leak rate and an underestimate of the ATP synthesis rate that occurred before oligomycin was added (Brand, 1990). With this proviso, Fig. 4B shows the estimates of the percentages of basal respiration used for ATP synthesis and proton leak within the cells. There was no difference between the haplogroups. The percentage of respiration used for ATP synthesis is the coupling efficiency, and, with a value of 85% of mitochondrial respiration, it was comparable to the coupling efficiency at intermediate respiration rates found in mitochondria isolated from the cells (Figs. 2 and 3), similar to the value of 80% measured previously using the parental cell line (Wu et al., 2007), and similar to values found in a range of other cell types (Brand, 2005).

Respiration rates in the presence of FCCP are not directly limited by ATP synthesis, and represent the maximal capacity of substrate oxidation by the cells under the conditions of measurement. In some cells this rate is equivalent to the maximum rate of the electron transport chain, but in others there is strong rate-limitation imposed by the supply of substrates to the mitochondria, particularly when ATP levels drop as a secondary result of uncoupling and substrates like glucose and fatty acids cannot be activated to glucose-6-phosphate and fatty acyl CoA. Fig. 4A shows that the FCCP-uncoupled respiration rates of A549 cybrids were initially high and rather variable, but then diminished with time, consistent with increasing limitation by substrate availability. There was no significant difference in uncoupled rates between haplogroups (Fig. 4B).

In summary, at the intact cell level we observed no significant functional differences between mitochondria within cybrids of haplogroup H and haplogroup T.

## 4 Discussion

There are published associations between mitochondrial haplogroups H and T and phenotypes that might be expected to have a strong bioenergetic component. However, our results show no significant bioenergetic differences in mitochondria with H or T-haplogroup mtDNAs in a constant nuclear background, at either the mitochondrial or the cellular level. We have previously shown that our methodology can pick up 10% differences in respiratory chain activity (Amo and Brand, 2007), so if haplogroup does affect mitochondrial bioenergetics, any effects must be very small. The lack of effect of haplogroup on mitochondrial coupling efficiency in intact cybrids shows that even if haplogroup affects mitochondrial proliferation or retrograde signalling, such changes do not affect coupling efficiency in the cells under basal conditions.

The strengths of our approach are (i) we used a direct empirical measurement, rather than inference from genetic and physiological traits, (ii) we separated the effects of mitochondrial haplogroup from any confounding effects of nuclear DNA background and (iii) we used a simple in vitro system with the powerful modular kinetic approach where the relevant variables could be tightly controlled and manipulated. However, our approach also has some weaknesses. (i) A small effect of mitochondrial haplogroup operating over hours or days might still be too small to be detected by biochemical experiments. (ii) We measured the bioenergetic capacity using isolated mitochondria and cybrid cells in simple defined media, but effects of mitochondrial haplogroups might only emerge under special conditions (for example, they might require triggers such as sepsis or energetic stress or might require particular nuclear genes to be expressed, and so be tissue-specific or suppressed in the lung carcinoma cell line we employed).

Our results fail to provide support for the hypothesis that the common European mitochondrial haplogroups H and T have significant effects on bioenergetics that cause the associations with survival after sepsis and sperm motility that have been reported. If such associations stand up to further scrutiny, they presumably operate through more subtle mechanisms than respiratory rates, mitochondrial coupling efficiencies or ATP supply.

## References

[bib1] Achilli A. (2004). The molecular dissection of mtDNA haplogroup H confirms that the Franco–Cantabrian glacial refuge was a major source for the European gene pool. Am. J. Hum. Genet..

[bib2] Amo T., Brand M.D. (2007). Were inefficient mitochondrial haplogroups selected during migrations of modern humans? A test using modular kinetic analysis of coupling in mitochondria from cybrid cell lines. Biochem. J..

[bib3] Baudouin S.V. (2005). Mitochondrial DNA and survival after sepsis: a prospective study. Lancet.

[bib4] Brand M.D. (1990). The proton leak across the mitochondrial inner membrane. Biochim. Biophys. Acta.

[bib5] Brand M.D., Brown G.C., Cooper C.E. (1995). Measurement of mitochondrial protonmotive force. Bioenergetics, a Practical Approach.

[bib6] Brand M.D. (1998). Top-down elasticity analysis and its application to energy metabolism in isolated mitochondria and intact cells. Mol. Cell. Biochem..

[bib7] Brand M.D. (2005). The efficiency and plasticity of mitochondrial energy transduction. Biochem. Soc. Trans..

[bib8] Brand M.D., Harper M.E., Taylor H.C. (1993). Control of the effective P/O ratio of oxidative phosphorylation in liver mitochondria and hepatocytes. Biochem. J..

[bib9] Brand M.D., Chien L.F., Diolez P. (1994). Experimental discrimination between proton leak and redox slip during mitochondrial electron transport. Biochem. J..

[bib10] Cann R.L., Stoneking M., Wilson A.C. (1987). Mitochondrial DNA and human evolution. Nature.

[bib11] Chagnon P., Gee M., Filion M., Robitaille Y., Belouchi M., Gauvreau D. (1999). Phylogenetic analysis of the mitochondrial genome indicates significant differences between patients with Alzheimer disease and controls in a French–Canadian founder population. Am. J. Med. Genet..

[bib12] Chomyn A. (1996). Platelet-mediated transformation of human mitochondrial DNA-less cells. Methods Enzymol..

[bib13] Dimauro S., Davidzon G. (2005). Mitochondrial DNA and disease. Ann. Med..

[bib14] Elson J.L., Turnbull D.M., Howell N. (2004). Comparative genomics and the evolution of human mitochondrial DNA: assessing the effects of selection. Am. J. Hum. Genet..

[bib15] Elson J.L. (2006). Does the mitochondrial genome play a role in the etiology of Alzheimer's disease?. Hum. Genet..

[bib16] Elson J.L., Turnbull D.M., Taylor R.W. (2007). Testing the adaptive selection of human mtDNA haplogroups: an experimental bioenergetics approach. Biochem. J..

[bib17] Herrnstadt C., Howell N. (2004). An evolutionary perspective on pathogenic mtDNA mutations: haplogroup associations of clinical disorders. Mitochondrion.

[bib18] King M.P., Attardi G. (1989). Human cells lacking mtDNA: repopulation with exogenous mitochondria by complementation. Science.

[bib19] Kivisild T. (2006). The role of selection in the evolution of human mitochondrial genomes. Genetics.

[bib20] Korshunov S.S., Skulachev V.P., Starkov A.A. (1997). High protonic potential actuates a mechanism of production of reactive oxygen species in mitochondria. FEBS Lett..

[bib21] Laird P.W., Zijderveld A., Linders K., Rudnicki M.A., Jaenisch R., Berns A. (1991). Simplified mammalian DNA isolation procedure. Nucleic Acids Res..

[bib22] Lambert A.J., Brand M.D. (2004). Superoxide production by NADH:ubiquinone oxidoreductase (complex I) depends on the pH gradient across the mitochondrial inner membrane. Biochem. J..

[bib23] Liu S.S. (1997). Generating, partitioning, targeting and functioning of superoxide in mitochondria. Biosci. Rep..

[bib24] Macaulay V. (1999). The emerging tree of West Eurasian mtDNAs: a synthesis of control-region sequences and RFLPs. Am. J. Hum. Genet..

[bib25] Mishmar D. (2003). Natural selection shaped regional mtDNA variation in humans. Proc. Natl. Acad. Sci. U. S. A..

[bib26] Montiel-Sosa F. (2006). Differences of sperm motility in mitochondrial DNA haplogroup U sublineages. Gene.

[bib27] Pereira L., Goncalves J., Goios A., Rocha T., Amorim A. (2005). Human mtDNA haplogroups and reduced male fertility: real association or hidden population substructuring. Int. J. Androl..

[bib28] Pereira L. (2007). No evidence for an mtDNA role in sperm motility: data from complete sequencing of asthenozoospermic males. Mol. Biol. Evol..

[bib29] Pike D.A. (2006). Phylogenetic networks for the human mtDNA haplogroup T. J. Gen. Genealogy.

[bib30] Protti A., Singer M. (2007). Strategies to modulate cellular energetic metabolism during sepsis. Novartis Found. Symp..

[bib31] Raule N., Sevini F., Santoro A., Altilia S., Franceschi C. (2007). Association studies on human mitochondrial DNA: methodological aspects and results in the most common age-related diseases. Mitochondrion.

[bib32] Reynafarje B., Costa L.E., Lehninger A.L. (1985). O_2_ solubility in aqueous media determined by a kinetic method. Anal. Biochem..

[bib33] Ruiz-Pesini E., Wallace D.C. (2006). Evidence for adaptive selection acting on the tRNA and rRNA genes of human mitochondrial DNA. Hum. Mutation.

[bib34] Ruiz-Pesini E. (1998). Correlation of sperm motility with mitochondrial enzymatic activities. Clin. Chem..

[bib35] Ruiz-Pesini E. (2000). Human mtDNA haplogroups associated with high or reduced spermatozoa motility. Am. J. Hum. Genet..

[bib36] Ruiz-Pesini E., Mishmar D., Brandon M., Procaccio V., Wallace D.C. (2004). Effects of purifying and adaptive selection on regional variation in human mtDNA. Science.

[bib37] Santoro A. (2006). Mitochondrial DNA involvement in human longevity. Biochim. Biophys. Acta.

[bib38] Taylor R.W., Turnbull D.M. (2005). Mitochondrial DNA mutations in human disease. Nat. Rev., Genet..

[bib39] Torroni A. (1997). Haplotype and phylogenetic analyses suggest that one European-specific mtDNA background plays a role in the expression of Leber hereditary optic neuropathy by increasing the penetrance of the primary mutations 11778 and 14484. Am. J. Hum. Genet..

[bib40] Wallace D.C. (2005). A mitochondrial paradigm of metabolic and degenerative diseases, aging, and cancer: a dawn for evolutionary medicine. Annu. Rev. Genet..

[bib41] Wallace D.C., Brown M.D., Lott M.T. (1999). Mitochondrial DNA variation in human evolution and disease. Gene.

[bib42] Wu M. (2007). Multiparameter metabolic analysis reveals a close link between attenuated mitochondrial bioenergetic function and enhanced glycolysis dependency in human tumor cells. Am. J. Physiol., Cell Physiol..

